# Rapid and MR-Independent *I*_K1_ Activation by Aldosterone during Ischemia-Reperfusion

**DOI:** 10.1371/journal.pone.0132592

**Published:** 2015-07-29

**Authors:** Joachim Alexandre, Thomas Hof, Paolo Emilio Puddu, René Rouet, Romain Guinamard, Alain Manrique, Farzin Beygui, Laurent Sallé, Paul Milliez

**Affiliations:** 1 CHU de Caen, Department of Cardiology, Caen, France; 2 Université de Caen Basse-Normandie, EA 4650 Signalisation, électrophysiologie et imagerie des lésions d'ischémie-reperfusion myocardique, Caen, France; 3 Department of Cardiovascular Sciences, Sapienza University, Rome, Italy; 4 Université de Caen Basse-Normandie, Medical School, Caen, F-14000, France; Inserm, FRANCE

## Abstract

In ST elevation myocardial infarction (STEMI) context, clinical studies have shown the deleterious effect of high aldosterone levels on ventricular arrhythmia occurrence and cardiac mortality. Previous *in vitro* reports showed that during ischemia-reperfusion, aldosterone modulates K^+^ currents involved in the holding of the resting membrane potential (RMP). The aim of this study was to assess the electrophysiological impact of aldosterone on *I*_K1_ current during myocardial ischemia-reperfusion. We used an *in vitro* model of “border zone” using right rabbit ventricle and standard microelectrode technique followed by cell-attached recordings from freshly isolated rabbit ventricular cardiomyocytes. In microelectrode experiments, aldosterone (10 and 100 nmol/L, n=7 respectively) increased the action potential duration (APD) dispersion at 90% between ischemic and normoxic zones (from 95±4 ms to 116±6 ms and 127±5 ms respectively, *P<0*.*05*) and reperfusion-induced sustained premature ventricular contractions occurrence (from 2/12 to 5/7 preparations, *P<0*.*05*). Conversely, potassium canrenoate 100 nmol/L and RU 28318 1 μmol/l alone did not affect AP parameters and premature ventricular contractions occurrence (except Vmax which was decreased by potassium canrenoate during simulated-ischemia). Furthermore, aldosterone induced a RMP hyperpolarization, evoking an implication of a K^+^ current involved in the holding of the RMP. Cell-attached recordings showed that aldosterone 10 nmol/L quickly activated (within 6.2±0.4 min) a 30 pS K^+^-selective current, inward rectifier, with pharmacological and biophysical properties consistent with the *I*_K1_ current (NPo =1.9±0.4 in control *vs* NPo=3.0±0.4, n=10, *P<0*.*05*). These deleterious effects persisted in presence of RU 28318, a specific MR antagonist, and were successfully prevented by potassium canrenoate, a non specific MR antagonist, in both microelectrode and patch-clamp recordings, thus indicating a MR-independent *I*_K1_ activation. In this ischemia-reperfusion context, aldosterone induced rapid and MR-independent deleterious effects including an arrhythmia substrate (increased APD_90_ dispersion) and triggered activities (increased premature ventricular contractions occurrence on reperfusion) possibly related to direct *I*_K1_ activation.

## Introduction

Aldosterone and mineralocorticoid receptor (MR) activation have an important pathophysiological role in cardiovascular events occurrence [1–4]. Furthermore, clinical studies have shown that high aldosterone levels at presentation or within the first days after the onset of ST elevation myocardial infarction (STEMI) are associated with short-term occurrence of major cardiovascular events (death, heart failure and life-threatening ventricular arrhythmias) [2–4] and that an early aldosterone blockade in STEMI context was associated with a reduction of life-threatening ventricular arrhythmias [5,6]. All these data strongly indicate that endogenous aldosterone can somehow promote ventricular arrhythmias and sudden cardiac death within a short timeframe; however the underlying exact mechanisms remain largely unknown, particularly whether these rapid effects are MR-mediated [7] or by the activation of distinct membrane receptors and ion channels [8].

During STEMI context, the “border zone” between normal and ischemic myocardium is a critical area suggested to promote the early emergence of automatic activity, focal or reentrant ventricular arrhythmias [9,10]. Recently, we found that aldosterone modulated cardiac ion currents involved in the holding of the resting membrane potential (RMP) during ischemia-reperfusion, with the probable implication of the sarcolemmal-K_ATP_ currents [11]. However, the involvement of other hyperpolarizing currents such as *I*_K1_ could not be excluded [12,13].

Therefore, the purpose of this study was, during this critical acute ischemia-reperfusion phase, to evaluate the electrophysiological impact of aldosterone on *I*_K1_ current using an *in vitro* model of rabbit right ventricle mimicking the “border zone” existing between normal and ischemic/reperfused areas and standard microelectrode technique, followed by cell-attached patches from freshly isolated rabbit ventricular cardiomyocytes.

## Methods

### In vitro model of “border zone”

#### Ethic statement

Experiments were carried out in strict accordance with the European Commission Directive 86/609/EEC for experimental care and with the recommendations in the Guide for the Care and Use of Laboratory Animals at the University of Caen. This study was also conducted with authorization for animal experimentation #14–98 from the local DDPP (Direction Départementale de la Protection des Populations) and the protocol was approved by the Committee on the Ethics of Animal Experiments of the University of Caen. Rabbits were housed at 18~22°C individually in stainless steel cages on racks equipped with automatic watering systems and free access to food, at the Animal Care Center of Caen University and received daily humane care. All procedures were humanely performed after general anesthesia has been induced, and all efforts were made to abolish suffering.

#### Materials

New Zealand white rabbits of either sex weighing 1.5 to 2.0 Kg were euthanized under anesthesia with sodium pentobarbital 125 mg/kg within the marginal vein. The hearts were quickly removed after thoracotomy and placed in a cardioplegic solution at room temperature for 30 min. One single thin standard longitudinal strip (16x8 mm) of the right ventricular free wall was pinned, endocardial surface upward, in a special dual perfusion chamber [14] (S1 Fig, volume of 5 mL) which is bisected by a thin latex membrane. This latex membrane is centrally perforated at its bottom to allow the single ventricular strip to be passed carefully through and so to be divided into two zones, called the Normal Zone (NZ) and the Altered Zone (AZ), respectively. This double compartment allowed the two parts of the same and single ventricular strip to be independently superfused at the rate of 3 mL/min with normal or modified Tyrode’s solution. The right ventricular base, near the tricuspid valve, was constantly placed in the NZ compartment. At the end of each experiment, absence of leakage between the 2 compartments was tested by a dye injection (methylene blue) in one of the 2 zones. Temperature at the level of the double chamber, including that of incoming fluids, was controlled and maintained to 36.5 ± 0.5°C by a circulating thermostat-controlled bath (Polystat 5HP, Bioblock, Illkirch, France).

#### Superfusion solutions and chemicals

Normal Tyrode’s solution was oxygenated with 95% O_2_ and 5% CO_2_ and kept at 37°C. The composition of this Tyrode’s solution was (in mmol/L): Na^+^ 135, K^+^ 4, Ca^2+^ 1.8, Mg^2+^ 1, H_2_PO_4_^-^ 1.8, HCO_3_^-^ 25, Cl^-^ 117.8, and glucose 11. The pH was 7.4. The cardioplegic solution used for dissection differed from normal Tyrode’s by higher glucose (from 11 to 55 mmol/L) and hyperkalemia (from 4 to 30 mmol/L). The modified, ischemia-simulating Tyrode’s solution differed from normal by elevated K^+^ concentration (from 4 to 12 mmol/L), decreased HCO_3_^-^ concentration (from 25 to 9 mmol/L) leading to a decrease in pH (from 7.4 to 7.0), a decrease in *P*O_2_ induced by replacement of 95% O_2_ and 5% CO_2_ by 95% N_2_ and 5% CO_2_, and withdrawal of glucose. All chemicals were purchased from Sigma Aldrich (Saint Quentin Fallavier, France). All drugs were first diluted in ethanol and then in Tyrode’s solution. The final concentration of ethanol for all drugs was near 0.1%. Controls were also treatted with ethanol 0.1% due to ethanol properties on *I*_K1_ currents [15].

#### Data Acquisition and Analysis

The data acquisition and analysis process has been described in detail previously [16]. The myocardial strips were stimulated at a frequency of 1 Hz via a bipolar silicon-coated steel wire electrode positioned either in the NZ or in the AZ. Stimulation was applied either in one or the other half of the muscle preparation with a home-built commutator. Rectangular pulses of 2 ms in duration and twice diastolic threshold intensity (around 2–2.5 V) were delivered by a programmable stimulator (SMP 310, Biologic, Grenoble, France). During the protocol, stimulation was stopped whenever sustained spontaneous arrhythmias occurred. Transmembrane potentials were recorded simultaneously in both myocardial regions by use of intracellular glass microelectrodes pulled from borosilicate filament tubes (GC 200F-15; Phymep, France) on a single barreled microelectrode puller (Narashige, distributed by OSI, France). Microelectrodes were filled with KCl 3 mol/L (tip resistance ranging from 10 to 30 MΩ) and coupled to Ag/AgCl microelectrode holders leading to a home-built double input stage of a high impedance capacitance-neutralizing amplifier. The two reference silver—silver chloride electrodes were positioned in the superfusate of each chamber, close to the preparation. The recordings were displayed on a memory dual-beam storage oscilloscope (Gould Instrument Systems Inc, USA). The following AP characteristics were automatically stored at identical time points for the two compartments and measured by a system of cardiac AP automatic acquisition and processing devices (DATAPAC, Biologic, Grenoble, France): RMP, AP amplitude (APA), AP duration at 90% of repolarization (APD_90_), and the maximal upstroke velocity (V_max_). During both simulated ischemia and reperfusion, spontaneous premature ventricular contractions were recorded and were classified in extrasystoles (1 to 3 spontaneous and continuous premature ventricular contractions), salvos (4 to 9 spontaneous and continuous premature ventricular contractions) and sustained activities (≥ 10 spontaneous and continuous premature ventricular contractions) [16]. To evaluate the ischemia-induced dispersion of repolarization, APD_90_ dispersion between NZ and AZ was calculated at the end of the simulated ischemia period as APD_90_ NZ- APD_90_ AZ at 30 min (at the end) of simulated ischemia. Whenever possible, the same impalement was maintained throughout the experiment; however, when it was lost, readjustment was attempted. If the readjusted parameters deviated ≤10% from the previous ones, experiments were continued; otherwise, they were discarded.

#### Experimental Protocol (S1 Fig)

We used similar experimental protocol than previously published [16]. During the 120-min stabilization period the right ventricular muscle was superfused with normal Tyrode’s solution and stimulated at a frequency of 1 Hz. Thereafter, a simulated ischemia was induced in 1 compartment (AZ) during 30 min by superfusion with the modified Tyrode’s solution (simulated ischemia period), while the other compartment (NZ) remained in normal conditions. At the end of the simulated ischemia, a 30-min reperfusion period was induced by perfusion of normal Tyrode’s solution in the AZ (reperfusion period). The NZ is perfused with normal Tyrode’s solution during all the experiment and so is not submitted to the simulated ischemia or to the reperfusion conditions. Unlike, the AZ is first submitted to 30 min of a simulated ischemia with a modified Tyrode’s solution and then to the reperfusion period during 30 min with the normal Tyrode’s solution. The administration of drugs began concomitantly with simulated-ischemia and continued until the end of the reperfusion period. There was no pretreatment period.

The preparations were randomly assigned to 7 groups: controls (n = 12), aldosterone 100 nmol/L (n = 7), 10 nmol/L (n = 7), aldosterone 10 nmol/L and potassium canrenoate 100 nmol/L (n = 7), aldosterone 10 nmol/L and RU 28318 1 μmol/L (n = 7), potassium canrenoate 100 nmol/L (n = 6), RU 28318 1 μmol/L (n = 6). The n-values given for microelectrode recordings are corresponding to the number of independent tissue preparations (or animal used). Indeed, each animal provided tissue used for only 1 experiment with standard microelectrode technique.

### Cell-attached patch clamp recordings

#### Isolation of rabbit ventricular myocytes

Rabbit ventricular cardiomyocytes were isolated by enzymatic digestion procedure modified from previously published protocol [17]. New Zealand’s white rabbits (1.5 to 2.0 kg) were euthanized by sodium pentobarbital 125 mg/kg within the marginal vein. Heparin 0.2 mg/kg was added in the same injection to avoid coagulation. The chest was open and the heart quickly removed and mounted on a Langendorff apparatus to be perfused retrogradely with a physiological isolation solution containing (in mmol/L): NaCl 130, KCl 5.4, NaH_2_PO_4_ 0.4, MgCl_2_ 1.4, CaCl_2_ 1, HEPES 10, glucose 10, taurine 20 and creatine 10 (pH at 7.4 with NaOH). When the coronary circulation had cleared of blood, perfusion was continued with Ca-free isolation solution (in which CaCl_2_ was replaced with 0.1 mmol/L EGTA) for 5 min, followed by perfusion for a further 6–7 min with isolation solution containing 0.8 mg/ml collagenase (type I; Worthington Biochemical, Lakewood, NJ), and 0.08 mg/ml protease (type XIV). Perfused solutions were maintained at 37°C and bubbled with 100% O_2_. Ventricles were then excised from the heart, minced, and gently shaken at 37°C in enzyme-containing solution supplemented with 1% bovine serum albumin. Cells were filtered from this solution at 5 min intervals, gently centrifuged and resuspended in isolation solution containing 1 mmol/L CaCl_2_. Cells were maintained in that solution to be used for patch-clamp recordings.

#### Patch-clamp measurements

Single-channel currents from cell-attached patches of freshly isolated ventricular cardiomyocytes were recorded under voltage clamp at room temperature (20–25°C). An Axopatch 200B (Axon Instruments, Sunnyvale, CA, USA) amplifier was used, controlled by a Pentium PC connected via a Digidata 1322A A/D converter (Axon Instruments), which was also used for data acquisition and analysis using Pclamp software (Axon Instruments). Isolated cells were placed in a bath solution containing (in mmol/L): NaCl 140, KCl 5.4, NaH_2_PO_4_ 0.4, CaCl_2_ 1 and HEPES 10 (pH 7.4). Pipette solution contained (in mmol/L): KCl 145, MgCl_2_ 1, CaCl_2_ 1 and HEPES 10 (pH 7.4). For estimation of ionic selectivity, the 145 mmol/L KCl in the pipette was replaced by 72.5 mmol/L KCl and 72.5 mmol/L K-glutamate or 72.5 mmol/L KCl and 72.5 mmol/L NaCl. Patch pipettes resistance was typically 3–4 MΩ. Single currents were filtered at 1 kHz and digitized at 5 kHz. NPo was determined by measuring the mean current I_mean_ for 30s windows and estimating the single channel current i at the corresponding voltage, according to the I/V relationship. We thus used the equation (I_mean_ = i NPo). All chemicals were from Sigma-Aldrich (Saint Quentin Fallavier, France). The n-values given for patch-clamp recordings are corresponding to the number of cells used. After each result, we specified the number of animals used for the cell recordings.

### Statistical Analysis

Results were expressed as mean ± standard error of the mean (SEM). In the two compartments, each cell served as its own control and significance of differences in absolute values was determined using analysis of variance (ANOVA) for repeated measures followed by Dunnet’s test as compared to initial values. Significance of differences between groups was determined using 2-factors ANOVA. The Fisher’s exact test was used for comparison of non-parametric categorical data. Paired or unpaired Student's *t*-test or non-parametric Wilcoxon test were used as appropriate to compare data from patch clamp recordings. In all analysis, *P<0*.*05* was considered statistically significant.

## Results

### In vitro model of “border zone”

#### Effects of Aldosterone and mineralocorticoid receptor blockers potassium canrenoate and RU 28318 on the AP Parameters

**Fig 1:**
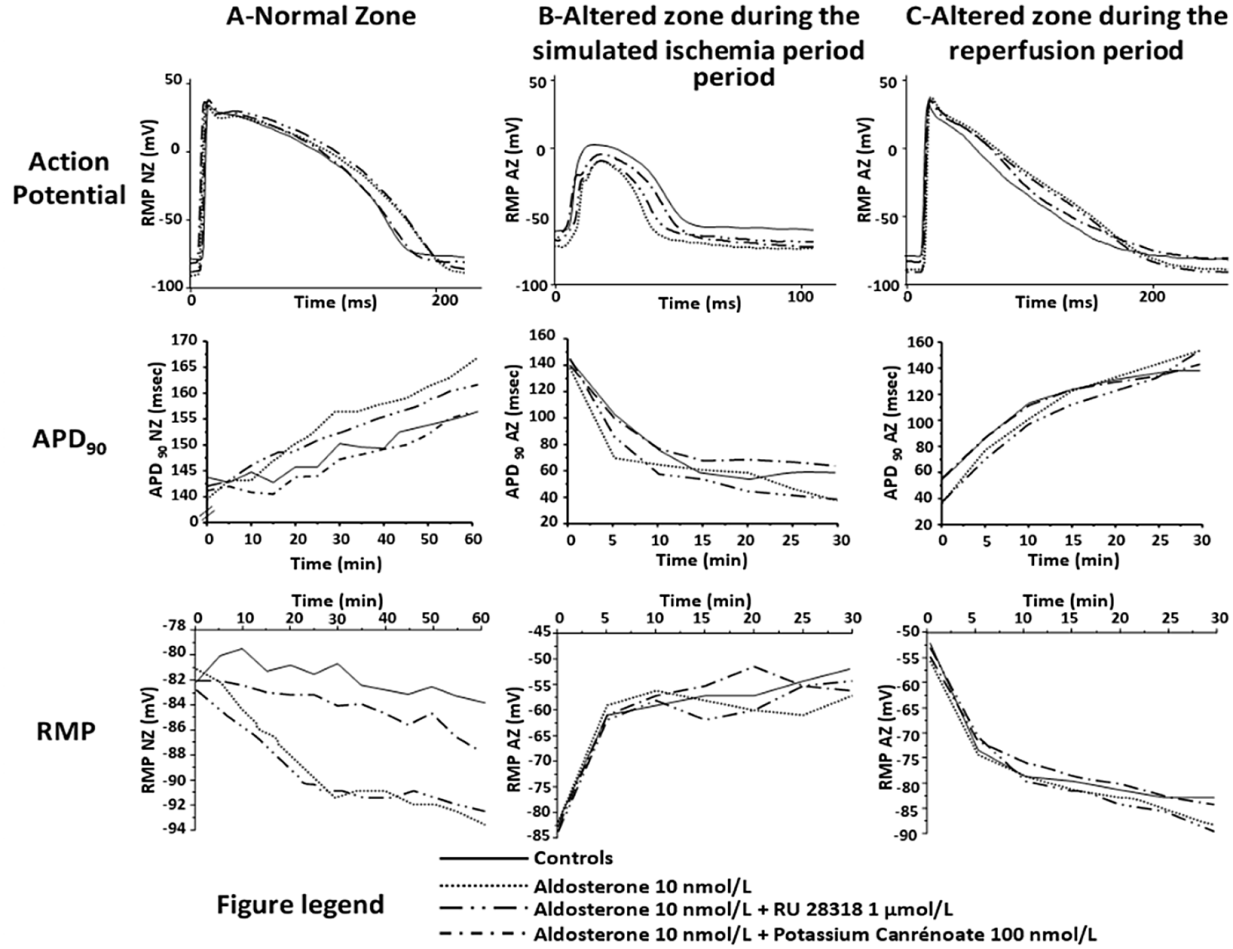
Effects of aldosterone and aldosterone + blockers (RU 28318 and potassium canrenoate) on APD_90_ and RMP, in the NZ (panel A) and in the AZ during both simulated-ischemia (panel B) and reperfusion periods (panel C). For sake of clarity and in order not to overload the figure, APD_90_ and RMP values are expressed in mean (and not in mean ± SEM). Concerning SEM and statistical significance, please refer to Tables 1 and 2.

In normoxic conditions (NZ), after 1 hour of superfusion, aldosterone was devoid of significant effects on V_max_ and on APA. Aldosterone 10 and 100 nmol/L (n = 7 respectively) induced a significant APD_90_ lengthening (from 156±5 ms to 165±3 ms and 175±6 ms respectively, *P<0*.*05*) and a change of RMP (hyperpolarization, from -84±2 mV to -92±7 mV and -93±5 mV respectively, *P<0*.*05*, Table 1, Fig 1). Potassium canrenoate and RU 28318 alone did not modify any AP parameter.

**Table 1:** Electrophysiological Effects of Aldosterone and Aldosterone blockers on AP parameters in Normoxic Conditions (NZ).

	Controls	Aldosterone		Potassium canrenoate	RU 28318	Potassium canrenoate 100 nmol/L + aldosterone 10 nmol/L	RU 28318 1 μmol/L + aldosterone 10 nmol/L
		10 nmol/L	100 nmol/L	100 nmol/L	1 μmol/L		
	(n = 12)	(n = 7)	(n = 7)	(n = 6)	(n = 6)	(n = 7)	(n = 7)
**RMP, mV**							
Initial	-82 ± 1	-81 ± 2	-82 ± 1	-81 ± 2	-83 ± 1	-82 ± 1	-83 ± 1
30 min	-81 ± 1	-90 ± 3^a^	-91 ± 2^a^	-82 ± 3	-84 ± 1	-84 ± 1	-86 ± 1^a^
60 min	-84 ± 2	-92 ± 7^a^	-93 ± 5^a^	-84 ± 1	-85 ± 2	-87 ± 2	-92 ± 1^a^
**APD**_**90**_ **, ms**							
Initial	143 ± 5	142 ± 2	141 ± 3	141 ± 4	144 ± 6	141 ± 3	142 ± 5
30 min	150 ± 4	156 ± 4	165 ± 4^a^	143 ± 3	146 ± 7	147 ± 4	152 ± 5
60 min	156 ± 5	165 ± 3^a^	175 ± 6^a^	149 ± 2	155 ± 5	156 ± 2	162 ± 3^a^

Values are mean ± SEM, RMP indicates resting membrane potentiel; APD_90_, action potential duration at 90% of repolarization.

^a^ P<0.05 vs controls values.

During the simulated ischemia period (Table 2), aldosterone did not significantly affect V_max_ and APA compared to controls. There was a significant APD decrease in both aldosterone 10 and 100 nmol/L groups (APD_90_, from 55±3 ms to 40±3 ms and 38±5 ms respectively, *P<0*.*05*). Potassium canrenoate 100 nmol/L significantly decreased Vmax (from 25±5 V/s to 13±3 V/s, *P<0*.*05*) without affecting RMP, APA and APD_90_. RU 28318 alone did not modify any AP parameter.

**Table 2:** Electrophysiological Effects of Aldosterone and Aldosterone blockers on AP parameters in Ischemic/Reperfused Conditions (AZ).

	Controls	Aldosterone		Potassium canrenoate	RU 28318	Potassium canrenoate 100 nmol/L + aldosterone 10 nmol/L	RU 28318 1 μmol/L + aldosterone 10 nmol/L
		10 nmol/L	100 nmol/L	100 nmol/L	1 μmol/L		
	(n = 12)	(n = 7)	(n = 7)	(n = 6)	(n = 6)	(n = 7)	(n = 7)
**RMP, mV**							
Initial	-82 ± 1	-81 ± 3	-81 ± 1	-82 ± 6	-83 ± 2	-83 ± 2	-84 ± 1
30 min	-52 ± 2	-55 ± 6	-54 ± 2	-56 ± 2	-59 ± 1	-55 ± 3	-53 ± 1
60 min	-82 ± 2	-89 ± 4^a^	-90 ± 3^a^	-83 ± 2	-83 ± 1	-83 ± 2	-89 ± 2^a^
**APD**_**90**_ **, ms**							
Initial	145 ± 5	140 ± 5	140 ± 4	139 ± 4	150 ± 8	141 ± 1	146 ± 6
30 min	55 ± 3	40 ± 3^a^	38 ± 5^a^	56 ± 4	56 ± 4	61 ± 4	40 ± 2^a^
60 min	143 ± 4	156 ± ^a^	160 ± 8^a^	139 ± 5	144 ± 10	148 ± 4	157 ± 2^a^

Values are mean ± SEM, RMP indicates resting membrane potential; APD90, action potential duration at 90% of repolarization.

^a^ P<0.05 vs controls values.

During the reperfusion period (Table 2), aldosterone 10 and 100 nmol/L induced a significant APD_90_ lengthening (from 143±4 ms to 156±5 ms and 160±8 ms respectively, *P<0*.05) and a RMP hyperpolarization (from -82±2 mV to -89±4 mV and -90±3 mV respectively, *P<0*.*05*). Potassium canrenoate and RU 28318 alone did not modify any AP parameter.

All the effects induced by aldosterone 10 nmol/L were successfully prevented in both NZ and AZ by adding potassium canrenoate 100 nmol/L but not by adding RU 28318 1 μmol/L (Tables 1 and 2, Fig 1).

#### Effects of Aldosterone, Potassium Canrenoate and RU 28318 on APD_90_ dispersion

**Fig 2:**
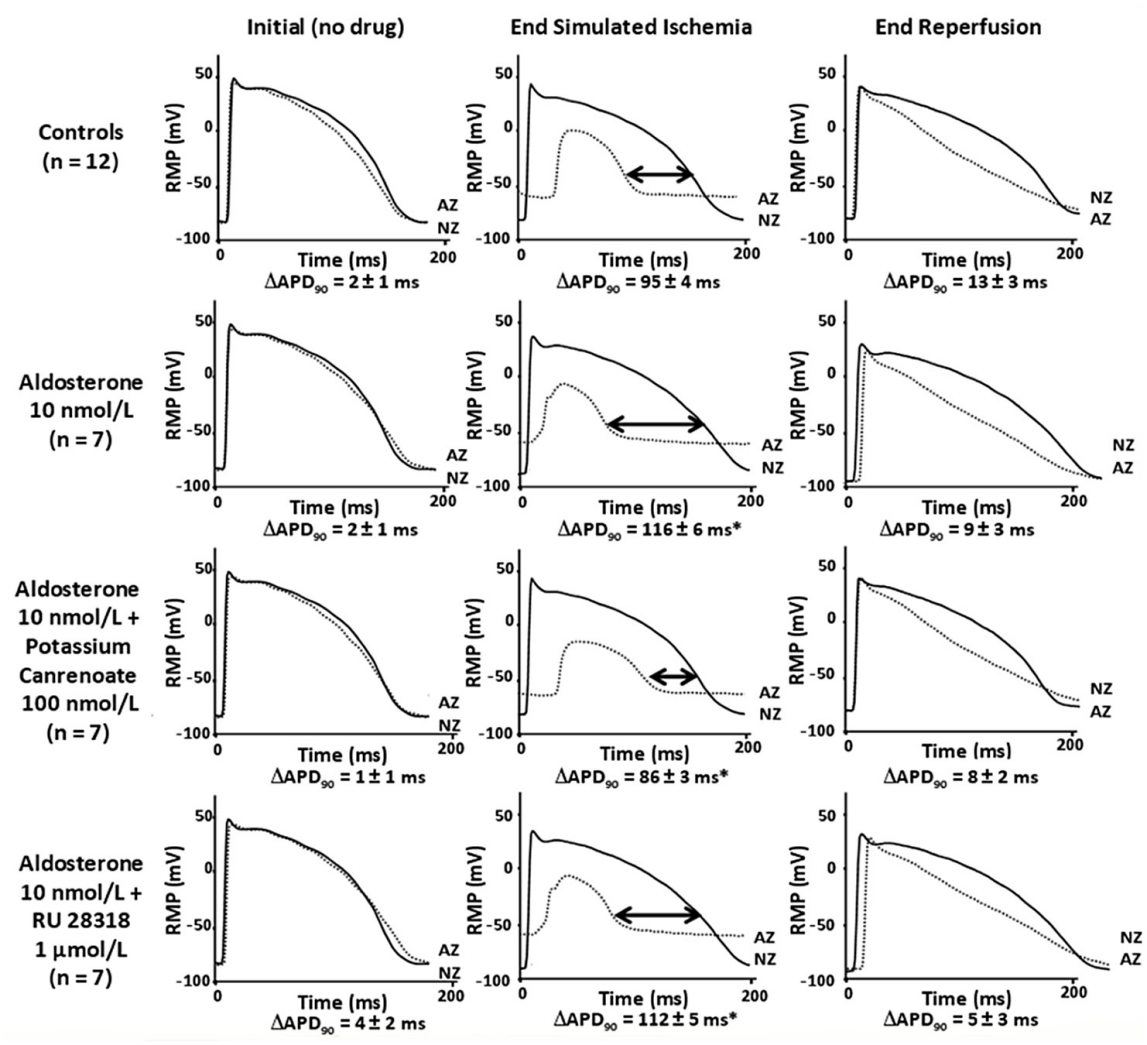
Representative AP recordings. Representative AP recordings obtained simultaneously in NZ and AZ in control and in presence of drugs, in same cell, in initial conditions (before initiation of ischemia and drug superfusion, left), at the end of simulated ischemia (at 30 minutes of experiment, middle) and at the end of reperfusion (at 60 minutes of experiment, right). APD_90_ dispersion between NZ and AZ (ΔAPD_90_) was measured at the end of the simulated ischemia period as APD_90_ NZ- APD_90_ AZ and is expressed as mean±SEM. Comparisons were made between control group and pharmacological groups. * *P<0*.*05* vs controls.

At baseline, in normal conditions, there was no significant APD_90_ dispersion between the two regions in all groups (Fig 2). Ischemic-like superfusion induced a significant increase in APD_90_ dispersion (*P<0*.*0001*) in all groups. APD_90_ dispersion was significantly increased by aldosterone 10 and 100 nmol/L (from 95±4 ms to 116±6 ms and 127±5 ms respectively, *P<0*.*05*). This increase of APD_90_ dispersion induced by aldosterone 10 nmol/L was prevented by adding potassium canrenoate 100 nmol/L but not by RU 28318 (respectively 86±3 ms and 112±5 ms *vs* 117±5 ms for aldosterone 10 nmol/L, *P<0*.*05*). Potassium canrenoate 100 nmol/L and RU 28318 1 μmol/L alone did not significantly affect APD_90_ dispersion.

#### Effects of Aldosterone, Potassium Canrenoate and RU 28318 on the Occurrence of Spontaneous premature ventricular contractions

**Fig 3:**
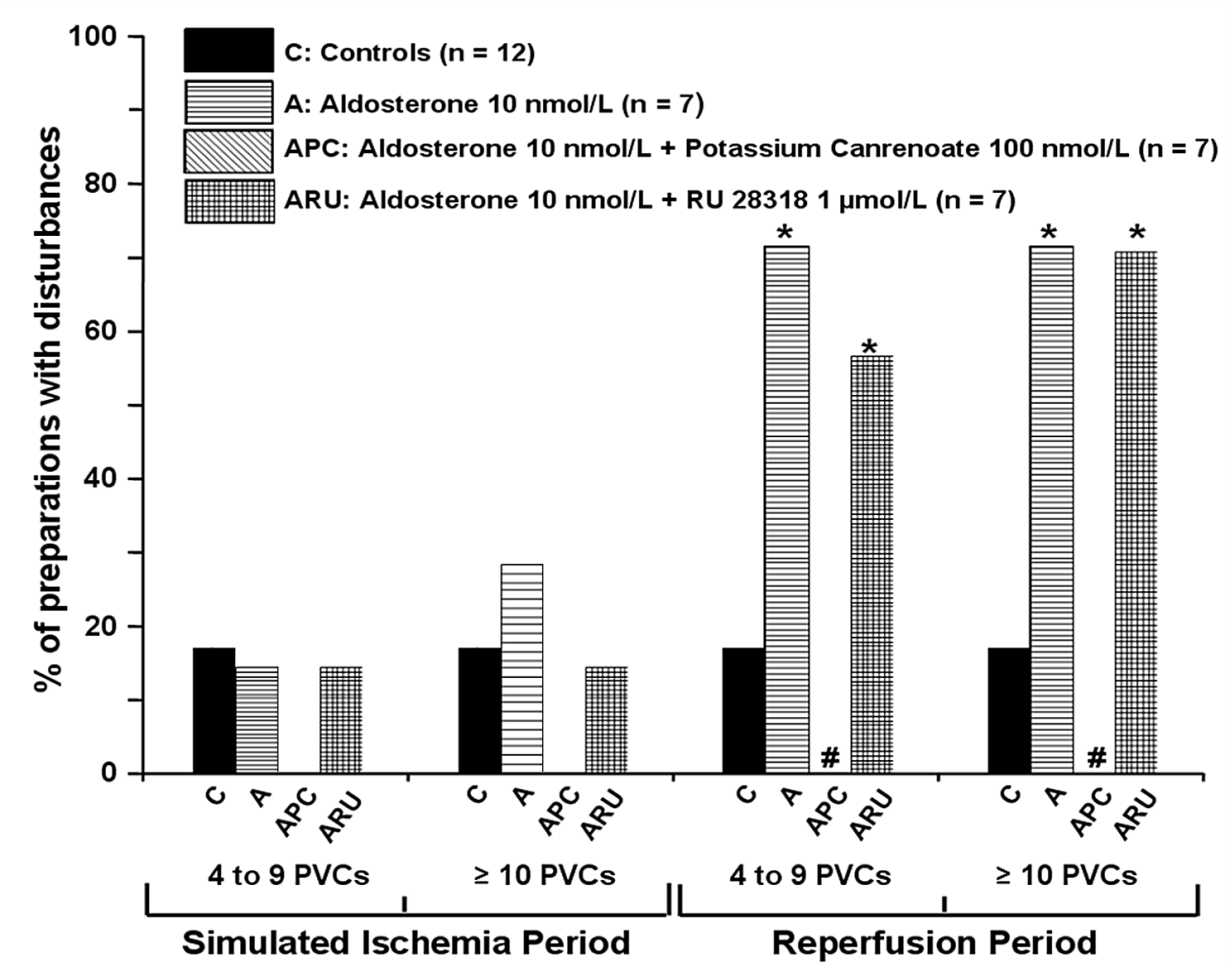
Effects of aldosterone and aldosterone + blockers (potassium canrenoate and RU 28318) on the incidence of spontaneous premature ventricular contractions during the 30-min of simulated ischemia and the 30-min of reperfusion. Data are expressed as percentage of preparations with disturbances. * P < 0.05 *vs* controls. ^#^ P < 0.05 between aldosterone and aldosterone + blockers.

At baseline, in normal conditions, spontaneous premature ventricular contractions were observed in none of the groups. Results are summarized in Table 3 and in Fig 3. During simulated ischemia period, aldosterone 10 and 100 nmol/L did not modify premature ventricular contractions occurrence.

**Table 3:** Effect of Aldosterone and Aldosterone blockers on the Incidence of Spontaneous premature ventricular contractions.

	Controls	Aldosterone 10 nmol/L	Aldosterone 100 nmol/L	Potassium canrenoate 100 nmol/L	RU 28318 1 μmol/L	Potassium canrenoate 100 nmol/L + aldosterone 10 nmol/L	RU 28318 1 μmol/L + aldosterone 10 nmol/L
	(n = 12)	(n = 7)	(n = 7)	(n = 6)	(n = 6)	(n = 7)	(n = 7)
**Simulated ischemia period**							
Extrasystoles (1–3 PVCs)	67	43	43	33	67	57	57
Salvos (4–9 PVCs)	17	14	43	17	33	0	14
Sustained activities (≥ 10 PVCs)	17	28	14	0	17	0	14
**Reperfusion period**							
Extrasystoles (1–3 PVCs)	92	100	86	83	83	86	57
Salvos (4–9 PVCs)	17	71^a^	71^a^	17	17	0	57^a^
Sustained activities (≥ 10 PVCs)	17	71^a^	71^a^	0	33	0	71^a^

Values are % of preparations with disturbances.

PVCs means premature ventricular contractions.

^a^ P<0.05 vs controls.

During reperfusion period, aldosterone 10 and 100 nmol/L significantly increased the occurrence of salvos and sustained premature ventricular contractions. This increase in arrhythmias was prevented by adding potassium canrenoate 100 nmol/L but not by RU 28318 1 μmol/L.

Potassium canrenoate 100 nmol/L and RU 28318 1 μmol/L alone did not significantly affect premature ventricular contractions occurrence.

#### Electrophysiological Effects of 1 and 10 μmol/L BaCL2 on the AP parameters in normal and Ischemic/Reperfused Conditions

We tested 1 and 10 μmol/L Ba^2+^, a selective *I*_K1_ blocker, in our “border zone” model. It induced a rapid AP extinction in the ischemic zone (lasting to the end of experiment) and a significant APD lengthening in the non-ischemic zone (Table 4).

**Table 4:** Electrophysiological Effects of 1 and 10 μmol/L BaCL2 on the AP parameters in normal (NZ, left panel) and Ischemic/Reperfused Conditions (AZ, right panel).

	NZ			AZ		
	Controls	BaCl_2_		Controls	BaCl_2_	
		10 μmol/L	1 μmol/L	**Controls**	10 μmol/L	1 μmol/L
	(n = 12)	(n = 3)	(n = 3)	(n = 12)	(n = 3)	(n = 3)
**RMP, mV**						
Initial	-82 ± 1	-79 ± 2	-82 ± 3	-82 ± 1	-79 ± 2	-84 ± 2
5 min	-80 ± 1	-73 ± 3	-83 ± 2	-60 ± 2	ND	-69 ± 8
10 min	-79 ± 1	-73 ± 6	-77 ± 3	-56 ± 2	ND	ND
30 min	-81 ± 1	-77 ± 2	-77 ± 2	-52 ± 2	ND	ND
60 min	-84 ± 2	-77 ± 3	-79 ± 5	-82 ± 2	ND	ND
**Vmax, V/s**						
Initial	279 ± 17	288 ± 22	282 ± 31	279 ± 17	243 ± 12	295 ± 24
5 min	260 ± 16	285 ± 14	265 ± 22	85 ± 13	ND	159 ± 34
10 min	235 ± 21	259 ± 12	238 ± 33	65 ± 11	ND	ND
30 min	255 ± 26	241 ± 15	246 ± 16	24 ± 5	ND	ND
60 min	251 ± 20	254 ± 23	285 ± 25	251 ± 20	ND	ND
**APD**_**90**_ **, ms**						
Initial	145 ± 5	143 ± 13	137 ± 8	145 ± 5	143 ± 8	138 ± 6
5 min	148 ± 5	149 ± 12	143 ± 10	104 ± 4	ND	96 ± 8
10 min	147 ± 5	151 ± 8	148 ± 5	77 ± 5	ND	ND
30 min	150 ± 4	157 ± 7	155 ± 7	55 ± 4	ND	ND
60 min	156 ± 5	184 ± 12	168 ± 11	143 ± 4	ND	ND

Values are mean ± SEM, RMP indicates resting membrane potential; Vmax, maximal upstroke velocity; APD_90_, action potential duration at 90% of repolarization;

NZ, normal zone; AZ, altered zone; ND, not determinable.

### Cell-attached patches results

**Fig 4:**
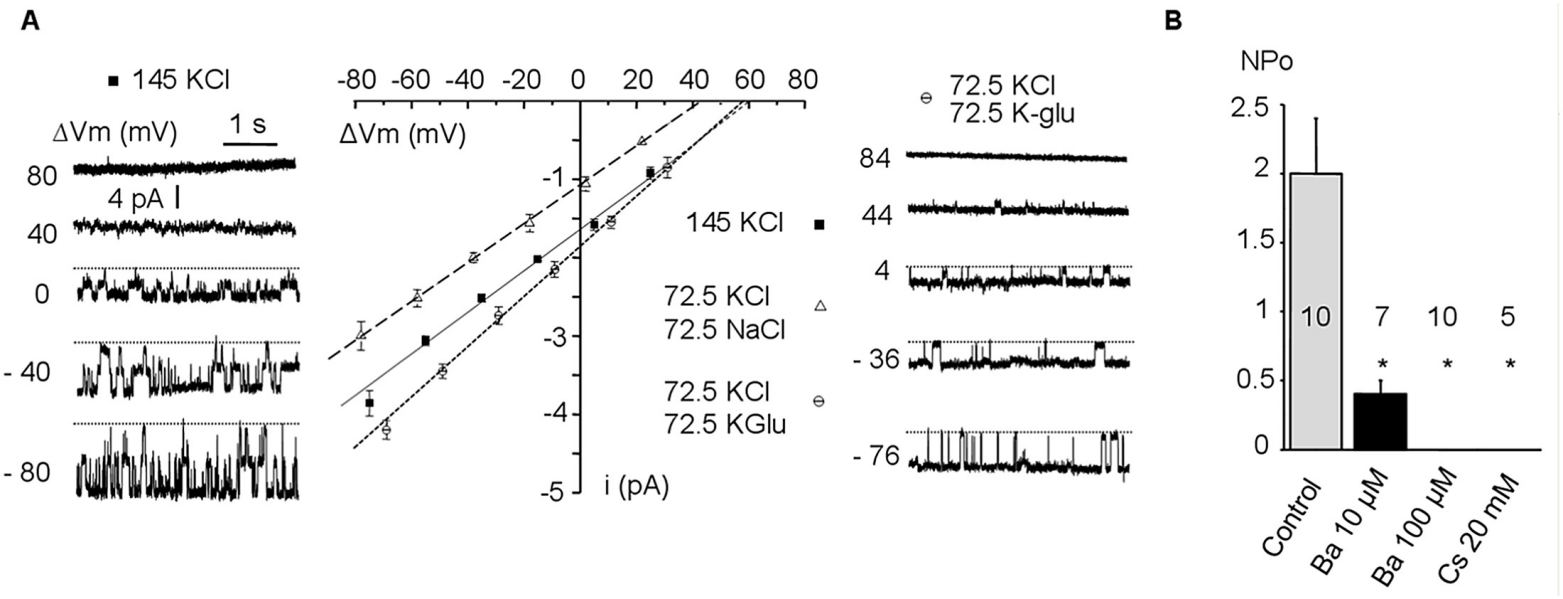
Cell-attached patches. Cell-attached patches from freshly isolated rabbit ventricular cardiomyocytes showing a 30 pS K^+^-selective channel, inward rectifier, with pharmacological and biophysical properties consistent with the *I*_K1_ current. A: Current voltage relationship (i/ΔVm) recorded in cell-attached conditions with 145 mmol/L KCl (black squares) or 72.5 mmol/L KCl and 72.5 mmol/L K-glutamate (open circles) or 72.5 mmol/L KCl and 72.5 mmol/L NaCl (open triangles) in the pipette. Typical single channel recordings are provided for several voltages for 145 mmol/L KCl (left pannel) or 72.5 mmol/L KCl and 72.5 mmol/L K-glutamate (right pannel). B: NPo of the basally active 30 pS channel in control condition (145 mmol/L KCl in the pipette) or with addition in the pipette of whether 20 mmol/L Cs^2+^, 10 or 100 μmol/L Ba^2+^. Numbers in bars are the numbers of experiments.

Only one type of spontaneously active channel was observed in 37/40 patches (92.5%), with 3.2±0.4 channels per patch, while the other patches did not show any activity. Current traces (Fig 4A) showed that its open probability was not affected by voltage, at least from -80 to +20 mV. No currents were detectable in the outward direction suggesting an inward rectification. The corresponding current—voltage relationship was linear for the inward currents with a slope conductance of 30±1 pS and a reversal potential (E_r_) estimated at +60.3±0.5 mV (n = 5, from 5 different rabbits) suggesting a K^+^ selectivity of the channel. The anion to cation selectivity was evaluated by substituting the 145 mmol/L KCl in the pipette solution by 72.5 mmol/L KCl and 72.5 mmol/L K-glutamate. It did not induce any significant change in the reversal potential (E_r_ = +57.6±2.5 mV; n = 6) indicating a lack of permeability for Cl^-^ (Fig 4A). The selectivity among cations was evaluated by substituting 72.5 mmol/L KCl in the pipette by the same concentration of NaCl. It induced a 17 mV left shift of the reversal potential (E_r_ = +44.4±1.8 mV; n = 5) in accordance with a K^+^ selectivity of the channel (Fig 4A). The addition of 20 mmol/L Cs^2+^ (non-selective *I*_K1_ blocker) or 100 μmol/L Ba^2+^ (selective *I*_K1_ blocker) in the pipette suppressed the 30 pS channel openings (normalized open probability NPo = 2.0±0.4, n = 10 in control *vs* NPo = 0±0, n = 5 and 10 respectively, *P<0*.*001*) while 10 μmol/L Ba^2+^ significantly reduced the NPo (0.4±0.1, n = 5, *P<0*.*05*) (Fig 4B). NPo was not affected by superfusing pinacidil 50 μmol/L (n = 7) or DNP 300 μmol/L (n = 4), excluding *I*_KATP_ as the determinant of the active channel recorded in our patches. Altogether, these results were compatible with the strong inward rectifier potassium current *I*_K1_.

**Fig 5:**
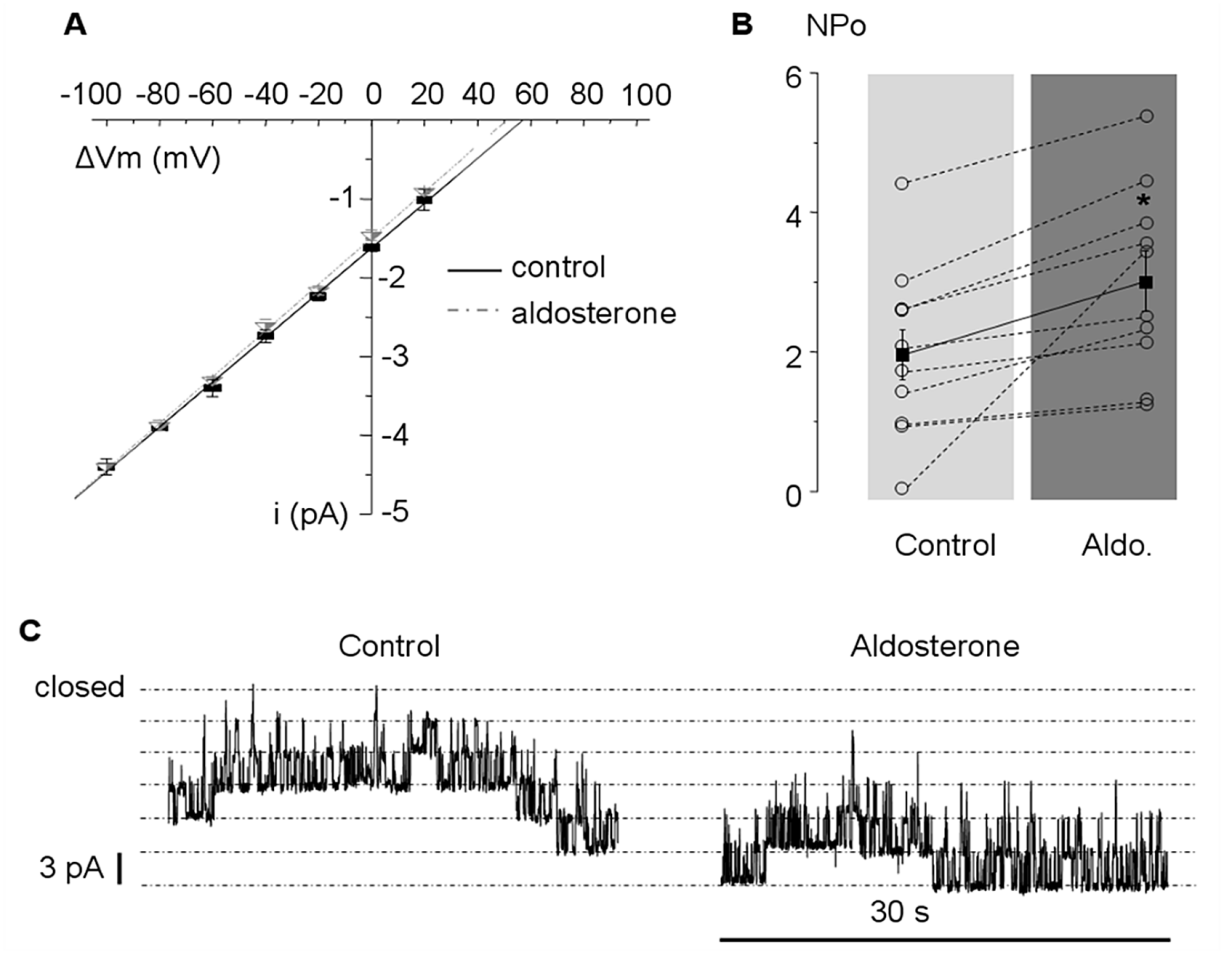
**A:** Current voltage relationship (i/ ΔVm) recorded in cell-attached condition with 145 mmol/L KCl in the patch pipette under control (dark squares and dark line) and after aldosterone superfusion (10 nmol/L) (open triangles and dotted line) **B:** Single channel NPo in control and during aldosterone superfusion in 10 cells. Open circle and dotted lines represent individual recordings. Mean is represented by dark squares and wide dark lines. **C:** Representative single channel current activity under control and under aldosterone superfusion (10 nmol/L) from the same patch membrane (ΔVm = -40 mV).

**Fig 6:**
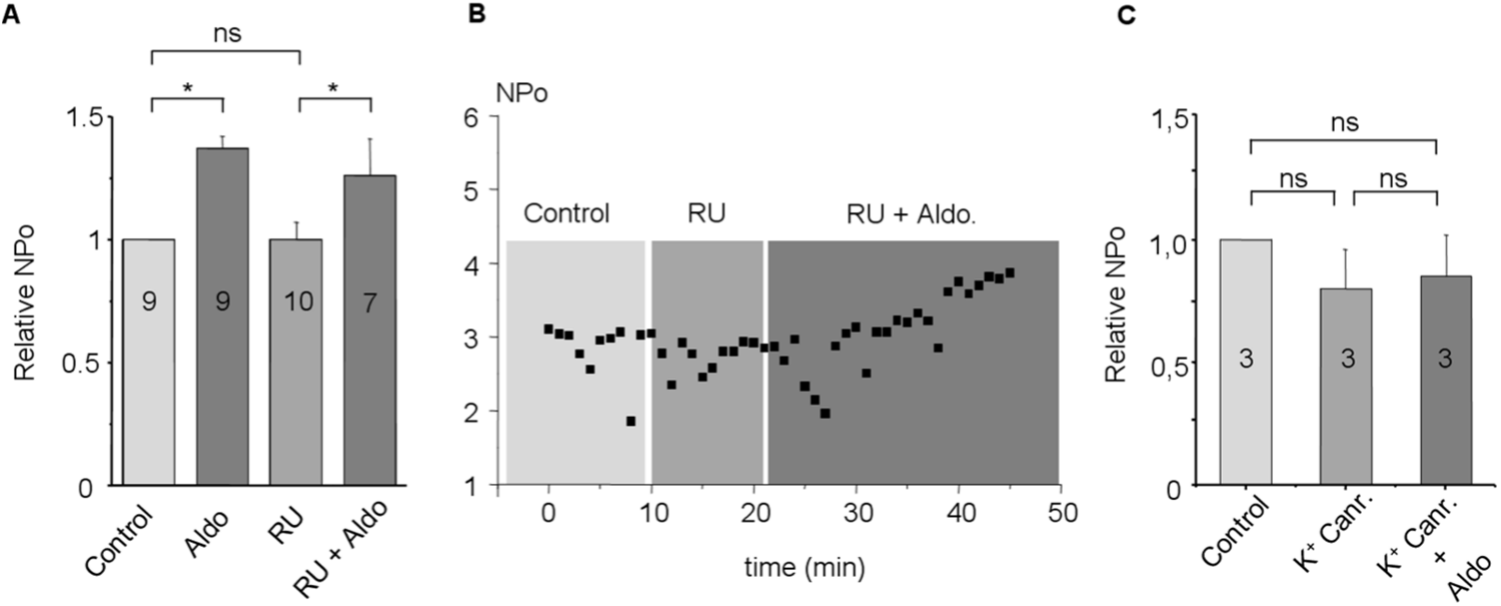
**A:** Single channel NPo under aldosterone superfusion (10 nmol/L), RU 28318 (1μmol/L) and aldosterone (10 nmol/L) with RU 28318 (1μmol/L) relative to the NPo in control **B:** Representative experiment showing the single channel NPo in function of the time under control followed by RU 28318 superfusion (1 μmol/L) and aldosterone (10 nmol/L) with RU 28318 (1 μmol/L) in the same patch membrane **C:** Single channel NPo under Potassium Canrenoate superfusion (100 nmol/L) and aldosterone (10 nmol/L) with Potassium Canrenoate (100 nmol/L) relative to the NPo in control. Numbers in bars are the numbers of experiments.*P<0.05; ns: non-significant.

Aldosterone 10 nmol/L was superfused during cell-attached recordings (holding potential = -40 mV). It produced in all patches a significant increase in channel NPo within 6.2±0.4 min until the end of the superfusion (1.9±0.4 in control *vs* 3.0±0.4 with aldosterone; *P<0*.*05*, n = 10) (Fig 5A and 5B) without affecting its single channel conductance (29.8±0.5 n = 6 in control vs 29.4±1.3 pS n = 5 under aldosterone superfusion) (Fig 5A). Increase in channel NPo persisted in presence of RU 28318 (NPo relative to the control = 1.4±0.05 under aldosterone alone, n = 9 *vs* 1.2±0.1 under aldosterone with RU 28318, n = 7, *P<0*.*05*) (Fig 6A and 6B). Superfusion of RU 28318 alone had no effect on NPo (NPo relative to the control = 1.0±0.1 under RU 28318 alone, n = 9) (Fig 6A and 6B). Potassium canrenoate superfusion with aldosterone prevented the aldosterone-induced increase in the NPo. 10 different rabbits were used to assess the aldosterone sensitivity. In our experimental conditions, no other aldosterone-activated channel type was observed.

## Discussion

The main findings of this study may be summarized as follows: 1. In standard microelectrode experiments, acute superfusion of aldosterone induced a RMP hyperpolarization, decreased ischemic APD_90_, increased APD_90_ dispersion and increased spontaneous premature ventricular contractions occurrence during the reperfusion period; 2. These effects suggested a modulation of a K^+^ current involved in the holding of the RMP; 3. Patch-clamp recordings unmasked an aldosterone-sensitive 30 pS K^+^-selective current, with pharmacological and biophysical properties compatible with the strong inward rectifier potassium current *I*_K1_; 4. Aldosterone effects persisted in presence of RU 28318, a specific MR antagonist, and were successfully prevented by potassium canrenoate, a non specific MR antagonist, in both microelectrode and patch-clamp recordings, thus indicating a MR-independent *I*_K1_ activation.

In our microelectrode experiments, aldosterone quickly increased the APD_90_ dispersion between ischemic and non-ischemic areas and therefore created an arrhythmia substrate [18]. This effect on dispersion of the repolarization of aldosterone was also demonstrated by Stambler *et al*. in a rat heart failure model treated with eplerenone [19]. In their study, eplerenone attenuated HF-related ventricular arrhythmia vulnerability by shorted prolongation of ventricular repolarization and repolarization dispersion. Human studies have also shown that endogenous aldosterone can be arrhythmogenic in patients with coronary artery disease without heart failure by promoting the lengthening of QTc intervals [20] and inducing repolarization heterogeneity [21], thus providing the substrate for the development of re-entrant ventricular arrhythmias [18]. Concomitantly, aldosterone also induced triggered activities by increasing spontaneous premature ventricular contractions occurrence. Interestingly, aldosterone increased spontaneous premature ventricular contractions occurrence only during the reperfusion period. This may have several explanations. It is known that ventricular arrhythmias occur preferentially within seconds or minutes of restoration of blood flow to previously ischemic myocardium or transiently (within few minutes) after a short and sudden ischemia [22]. Moreover, we recently found that aldosterone could also modulate K_ATP_ channels [11] which are known to favor ventricular arrhythmias occurrence during reperfusion *via* an inability to prevent cell membrane phospholipids injury [23,24]. Finally, these triggered activities could be linked to cellular Na^+^/Ca^2+^ overload, thus aldosterone increases intracellular Ca^2+^ levels [25].

These deleterious effects of aldosterone observed in standard microelectrode experiments seemed to involve K^+^ currents involved in the holding of the RMP and our patch-clamp recordings showed an aldosterone-sensitive inward rectifier K^+^ current. Cardiomyocytes are known to hold three inward rectifier K^+^ currents [13]: *I*_K1_ that is more prominent in ventricles compare to atria, *I*_K,Ach_ that inversely is prominent in atria, and *I*_K,ATP_ that exhibits a weaker inward rectification than others [12]. In our standard microelectrode experiments, we could not distinguish the own impact of each current and maybe several of these currents were responsible for the observed results. We recently shown that aldosterone also modulates K_ATP_ channels [11]. However, in the actually patch-clamp recordings, pinacidil and DNP did not increase NPo of the aldosterone-sensitive inward rectifier K^+^ current, thus excluding *I*_KATP_ identity. *I*_KAch_ was discarded due to its predominance in atria and therefore the very low probability to register this current in ventricles [13]. In cardiac tissue, the molecular identity of *I*_K1_ currents is Kir2.x with a conductance range from 10 to 40 pS [12]. Because Kir2.1 was reported to be functionally expressed in ventricular rabbit myocytes and exhibits a single channel conductance of 30 pS, it more likely corresponds to the channel recorded in our preparation [12]. While *I*_K1_ is mostly considered to be active during the hyperpolarization of the membrane, it also participates in the AP, immediately after upstroke and during phase 3 repolarization, providing a repolarizing outward current [26]. Therefore, modulation of *I*_K1_ would likely have a profound effect on cardiac excitability and arrhythmogenesis [27]. The role of Kir2.1 in ventricular arrhythmia vulnerability has been highlighted by the recently described Andersen’s syndrome [28], in the guinea pig heart model of ventricular fibrillation [29], in a transgenic mouse model [30] and recently in human KCNJ2 mutation [31]. Therefore, *I*_K1_ activation during ischemia-reperfusion context may have a deleterious impact on ventricular arrhythmogenesis.

Unfortunately, in our standard microelectrode experiments, it was not possible to highlight a possible protective role of *I*_K1_ current blockade by Ba^2+^ on deleterious effects induced by aldosterone. It was demonstrated that 10 μmol/L Ba^2+^ partially but selectively inhibit *I*_K1_ [32]. At this concentration, Ba^2+^ does not affect *I*_Kr_, *I*_Ks_, *I*_to_ or *I*_KATP_ but depresses selectively *I*_K1_ by about 60% [33]. When we tested this concentration in our microelectrode experiments, it induced a rapid AP extinction in the ischemic zone (lasting to the end of experiment) and a significant APD lengthening in the non-ischemic zone (Table 4). 1 μmol/L Ba^2+^ had the same effects and we did not test lower concentrations because of the loss of *I*_K1_ inhibition that would have resulted. This ischemic AP extinction may be explained by the strong ischemia and depolarization induced by our simulated ischemia added to Ba^2+^ effects. Interestingly, concomitantly to this "non-viable" ischemic AP, we did not observe premature ventricular contractions. Clearly, there are major differences between isolated cells and tissues which may well account for these discepancies.

Our results might indicate that aldosterone induced arrhythmogenesis by a MR-independent *I*_K1_ activation during myocardial ischemia-reperfusion context. Some studies have shown that deleterious effects of increased concentrations of aldosterone may be related to slow genomic mechanisms (within weeks), through activation of specific cytosolic MR [7] and to rapid non-genomic mechanisms (within minutes to days), through activation of membrane receptors and ion channels [8,34]. Wehling and colleagues showed that aldosterone at nanomolar concentrations produced rapid, nongenomic and MR-independent effects in a variety of tissues [35]. However, evidence has been forthcoming that these rapid nongenomic effects of aldosterone on cardiomyocytes are mediated *via* the classical MR [36]. In recent clinical trials [5,37], the deleterious impact of aldosterone on sudden cardiac death and ventricular arrhythmias occurred within a short timeframe, too short, apparently, to be attributed to MR-dependent mechanisms, in accordance with our results. In our experiments, this rapid and deleterious *I*_K1_ activation induced by aldosterone superfusion, in both standard electrodes and patch-clamp recordings, persisted in presence of RU 28318 and were successfully prevented by potassium canrenoate. Although the beneficial effects of MR antagonists are now well established in clinical practice [6,38–40], there is some debate about their specificity, particularly regarding potassium canrenoate and spironolactone, which can also antagonize the glucocorticoid and progesterone receptors; in contrast, RU 28318 specifically antagonizes the MR [41]. Although K^+^ currents, particularly *I*_K1_ and *I*_KATP_, seem to be responsible for a critical part of aldosterone arrhythmogenesis in our standard microelectrode experiments, they are probably not the only currents involved. Indeed, the RMP was shifted towards more negative values (Table 1), while APD_90_ was prolonged. Neither of these effects can be explained by an increase in solely *I*_K1_ and *I*_KATP_. An increased *I*_K1_ alone may have some impact on RMP, but unlikely to the extent as shown here [33]. Furthermore, increased *I*_K1_ and *I*_KATP_ add to the repolarization capacity and would shorten the AP [42]. Therefore, it is highly probable that additional ionic currents were affected by aldosterone treatment in our “border zone” experiments. Activation of aldosterone/MR deeply modulates cardiac electrical activity and leads to ventricular arrhythmias. The increase of MR expression directly impact on T-type calcium channels activity, decrease *I*_*Kr*_ activity and impaired the activity of the ryanodine receptor in mouse atrial HL1 cells [43]. *Ex vivo*, aldosterone increases the L-type calcium current in neonatal rat cardiomyocytes [44,45]. *In vivo*, cardiomyocyte-specific MR overexpression in transgenic mice induces ionic remodeling with a decrease of the outward potassium current (*I*_*to*_) activity and an increase in L-type Ca channel activity which is associated with ventricular extrasystoles and an increase in sensitivity to the triggering of ventricular arrhythmia [46]. Aldosterone is also known to block *I*_Ks_ and *I*_Kr_ currents and increase *I*_Na_ and *I*_K,ATP_ current [11,47,48]. These currents are probably mainly responsible for AP prolongation and could also participate in the pro-arrhythmic effects induced by aldosterone. Interestingly, we did not observed RMP hyperpolarization during the simulated ischemia period. It is probably due to a competition with the strong depolarization induced by the modified ischemia-simulating Tyrode solution. Finally, Kir2 have pH dependence depending on the isoforms. This is especially relevant due to isoform differences between species [49]. As ischemia-reperfusion and aldosterone both affect tissue pH, this could explain that the RMP hyperpolarization induced by aldosterone in our experiments was not observed in other preparations (mouse ventricular myocytes) [47] and might be related to species-specific differences.

## Study Limitations

The study was performed in rabbit myocardium because although rabbit myocardium differs from human myocardium, the main ionic currents involved are qualitatively similar (compared to rat or mice myocardium) [50] and because our *in vitro* model of "border zone" requires the use of a right ventricular strip measuring at least 13–14 mm (mean rabbit right ventricular strip length of 16x8 mm used in our preparations). We did not use left ventricular myocardium because of its excessive thickness not compatible with our experimental model associated with the risk of poor perfusion in the center of the tissue that could cause ischemia. Unfortunately, we were unable to inhibit *I*_K1_ in our “border zone” model and therefore we cannot directly link the arrhythmogenicity observed in this model to a role of *I*_K1_ (Table 4). This right ventricular *in vitro* model could induce an intramural ischemia since the tissue is superfused rather than perfused. This, added to the fact that we practiced recordings at 37°C, could explain the non-significant APD_90_ and RMP time-variation observed during experiments in the NZ of the control group (Fig 1). *I*_*K1*_ current activation is known to decrease with temperature [51,52] so we could therefore suppose that in our patch-clamp experiments conditions (room temperature), the aldosterone-induced *I*_*K1*_ current activation could be underestimated. Whereas RU 28318 is a specific and selective MR antagonist, potassium canrenoate, the active metabolite of spironolactone, is also able to block glucocorticoid and progesterone receptors, along with other ion currents [48]. Potassium canrenoate also has a major effect on cardiac excitability and inotropism [53,54]. These properties could introduce a bias in our results. Finally, we used RU 28318 as a specific MR antagonist and not eplerenone because when we performed our experiments, eplerenone was unfortunately not freely available. As our experiments seem to indicate a MR-independent pathway, the experiments using RU 28318 cannot be strictly considered as their own positive controls.

## Conclusion

In this *in vitro* model mimicking the “border zone” existing between normal and ischemic/reperfused regions, aldosterone induced deleterious electrophysiological effects including an arrhythmia substrate (increasing APD_90_ dispersion) and triggered activities (increasing premature ventricular contractions occurrence on reperfusion). In microelectrode experiments, these effects might be related with the modulation of K^+^ currents involved in the holding of the RMP. Our patch-clamp recordings showed an aldosterone-sensitive 30 pS K^+^-selective channel, with pharmacological and biophysical properties consistent with the strong inward rectifier K^+^ current *I*_K1_. These deleterious effects persisted in presence of RU 28318, a specific MR antagonist, and were successfully prevented by potassium canrenoate, a non specific MR antagonist, in both microelectrode and patch-clamp recordings, thus indicating a MR-independent *I*_K1_ activation. This new highlighted mechanism is in line with current clinical data [2,4,5] and could help to understand underlying mechanisms explaining the increase of ventricular arrhythmia and sudden cardiac death occurrence concomitant with high aldosterone levels during STEMI.

## Supporting Information

S1 Fig The double compartment tissue bath used for the experiments. The volume chamber of 5 ml enabled the right ventricular strips (slightly longer in rabbits) to be gently passed under the latex membrane in order to have one portion superfused with normal Tyrode’s and the adjacent portion with the altered, ischemia-simulating solution.
(TIF)

S2 Fig Experimental protocol. NZ means Normal Zone; AZ altered Zone.
(TIF)
