# Supplementary figures and images for: Rapid and MR-Independent I K1 Activation by Aldosterone during Ischemia-Reperfusion
Source: PLoS One. 2015 Jul 29;10(7):e0132592. doi: 10.1371/journal.pone.0132592 (PMC4519293; doi:10.1371/journal.pone.0132592)

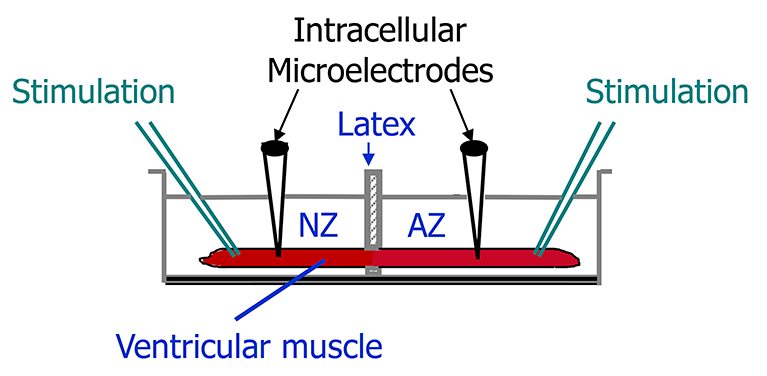

Supplement: S1 Fig — The volume chamber of 5 ml enabled the right ventricular strips (slightly longer in rabbits) to be gently passed under the latex membrane in order to have one portion superfused with normal Tyrode’s and the adjacent portion with the altered, ischemia-simulating solution. (TIF) [file pone.0132592.s001.tif]

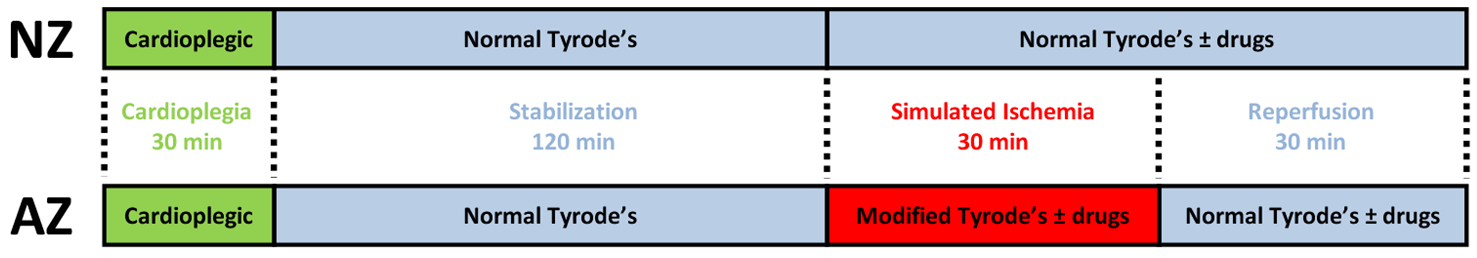

Supplement: S2 Fig — NZ means Normal Zone; AZ altered Zone. (TIF) [file pone.0132592.s002.tif]
